# The increase in child obesity in Switzerland is mainly due to migration from Southern Europe – a cross-sectional study

**DOI:** 10.1186/s12889-021-10213-0

**Published:** 2021-01-29

**Authors:** Urs Eiholzer, Chris Fritz, Anika Stephan

**Affiliations:** Center for Paediatric Endocrinology Zurich (PEZZ), Moehrlistrasse 69, CH-8006 Zurich, Switzerland

**Keywords:** Overweight, Obesity, Migration, Children, BMI

## Abstract

**Background:**

Novel height, weight and body mass index (BMI) references for children in Switzerland reveal an increase in BMI compared to former percentile curves. This trend may be the result of children with parents originating from Southern European countries having a higher risk of being overweight compared to their peers with parents of Swiss origin. We examined the association of generational, migration-related and socioeconomic factors on BMI in Switzerland and expect the results to lead to more targeted prevention programs.

**Methods:**

From contemporary cross-sectional data, we calculated subgroup-specific BMI percentiles for origin. Results for children of Swiss origin were compared with historical BMI data from Zurich. We tested for the association of overweight and obesity with origin and compared the distributions of BMI percentile ranks. Logistic regression analyses were applied to predict probabilities of being overweight or obese by origin and the Swiss neighborhood index of socioeconomic position (SSEP).

**Results:**

Compared to the BMI from two generations ago, the newly calculated BMI increased only slightly for children with both parents from Switzerland; 1.2% of these girls and 1.6% of these boys are obese. In the Swiss population, 13% of the children have parents from Southern Europe and the proportion of obesity is 57 and 42% in these boys and girls, respectively. Their BMI medians correspond to those of their parents’ countries of origin. For the probability of being overweight or obese, the SSEP differences are less important than the status of origin.

**Conclusion:**

We identified children with both parents from Southern Europe as the main influence driving the increase in BMI in Switzerland over the past 50 years. A differentiated consideration of the proportions of various migrant groups within cross-sectional samples is essential when monitoring BMI. Ignoring fluctuations can lead to false conclusions.

## Background

In Europe, the prevalence of obesity in children and adolescents has been rising since the 1980s [1]. While the proportions of overweight and obese children have more or less stabilized or decreased in Northern and Central Europe between 1999 and 2016, these numbers are still increasing for children from Southern Europe that have the highest prevalence rates [1]. Recent data collected from school-aged children in Switzerland between 2002 and 2017 shows a weak but significantly declining trend in overweight and obesity [2].

Demographic changes through migration are likely to influence the prevalence of obesity. As 25% of the people residing in Switzerland today are migrants, a differentiated view regarding national origin is essential to understand the variations in the prevalence of overweight and obesity. The immigrant population profile in Switzerland has diversified from mainly unqualified Spanish and Italian workers in the 1960s and immigrants from Portugal and the former Yugoslavia in the 1990s towards a large proportion of tertiary educated European Union citizens within the last twenty years. Currently, the largest foreign populations in Switzerland are from Italy followed by Germany, Portugal, and France.

In 2019, new height, weight and body mass index (BMI) references were published for children in Switzerland, including those with parents of non-Swiss origin [3]. The comparison of today’s children with the former percentile curves of Prader et al. two generations ago [4] showed an increase in BMI. Further, it was shown that children with parents originating from Southern Europe have a 2.5-fold higher risk of being overweight over children with both parents of Swiss origin [3].

Switzerland is a small country with an obligatory residence register and an up-to-date migration statistics database, which applies for all 26 cantons. Therefore, it is possible to study the influence of migration on weight in greater detail. In this paper, we examine the generational, migration-related and socioeconomic factors associated with the prevalence of overweight and obesity of children in Switzerland.

## Methods

For this study, we used cross-sectional data collected from boys and girls aged between 0 to 20 years living in Switzerland; the methods used for data collection and data cleaning processes have already been described in detail [3]. Briefly, our sample comprised prospectively measured data of 12,543 children and adolescents collected at pediatric practices and schools, which we refer to as our “contemporary core sample” throughout the remainder of this article. These data were originally supplemented with three external retrospective data sets that were excluded for the purposes of this study because detailed information outlining the origin of parents was not documented [3].

From our contemporary core sample, the following variables of BMI, age, sex, Swiss neighborhood index of socioeconomic position (SSEP), origin of the mother and origin of the father were used for analysis. Origin of the mother/father indicates the region of birth. Countries, if not themselves coded as a region, were not documented. Regions were defined as: (1) Switzerland, (2) Northern/Central Europe: Germany, Austria, France, Belgium, Netherlands, Luxembourg, Sweden, Finland, Norway, Denmark, (3) Italy, Portugal, Spain, (4) Balkan region: Albania, Bosnia-Herzegovina, Bulgaria, Greece, Kosovo, Croatia, Moldova, Montenegro, Northern Macedonia, Romania, Serbia, Slovenia, (5) Turkey, (6) other countries. Based on the combination of each parent’s origin we defined the following categories for origin: (1) Switzerland, (2) Northern/Central Europe (3) Italy, Portugal, Spain, (4) Balkan region (5) Turkey, (6) one parent from Switzerland, or (7) other: parents not from the same region/one or both from other regions. In this publication, we applied the term “Southern Europe” to encompass all three defined origin categories of Italy, Portugal, Spain as well as the Balkan region and Turkey. The SSEP is an area-based index expressed as median per postal code area and combines information on income, education and occupation, whereby data are based on the structural survey of 2012–2015 [5].

Sex-specific BMI percentile curves by origin were calculated with the LMS method [6]. The age scale was not transformed. For the evaluation of model fit, we used Q-tests [7]. All notation for percentiles employed the term “P” and the number indicating the nth percentile; for example, the 50th percentile was labelled “P50”.

We compared P10, P50 and P90 for children with both parents of Swiss origin to children two generations ago (also known as the “Prader sample” for the purposes of this work) from the respective smoothed BMI-for-age percentiles of the working group of Prader [4]. Differences were calculated in kilograms per meters squared (kg/m^2^) and in kilograms (kg) for a boy or girl of P50 height-for-age.

Prevalence of overweight including obesity and of obesity alone, were calculated using the International Obesity Task Force (IOTF) cut-offs. These cutoffs are defined by the percentiles at BMI values of 25 kg/m^2^ and 30 kg/m^2^ at the age of 18 years throughout childhood and adolescence and were derived from six large data sets from Brazil, Great Britain, Hong Kong, the Netherlands, Singapore, and the United States [8]. We applied a χ^2^-test for association between IOTF category (non-overweight, overweight, obese) and origin. Additionally, we calculated the distributions of origin within the total sample and within individuals categorized as overweight (including obese) and obese.

For analyzing the association with origin, we calculated the sex-specific empirical cumulative distribution functions of percentile ranks based on the new Swiss BMI percentiles [3] and plotted the deviations by origin. This was made with the intention to also evaluate differences in the lower and middle ranges of BMI without focusing only on the prevalence of overweight and obesity. To include the Prader sample in this plot, we calculated z-values for the new Swiss BMI P3, P10, P25, P50, P75, P90 and P97 with respect to the age- and sex-specific Prader means and standard deviations, and transformed z into percentile ranks. Percentile ranks were then averaged over age.

To visualize the fit of the BMI percentiles for the groups of children with parents of non-Swiss origin, we plotted their raw data and group-specific P10, P50 and P90 over the new P10, P50 and P90 for Switzerland [3], and added available percentiles of the respective European countries or regions (i.e. Italy [9], Spain [10], Portugal [11], Albania [12], Macedonia [13], Serbia [14], Turkey [15–19], Germany [20], and Austria [21]).

We predicted probabilities of being overweight or obese by applying logistic regression for children dependent on their parents’ origin and SSEP [3]. Origin was dichotomized (‘both parents from Switzerland’ versus ‘both parents from Southern Europe’) and the sample included only children of ≥8 years.

For LMS modelling, we used LMSchartmaker Pro version 2.54 (Medical Research Council, Cambridge, UK). For logistic regression analyses, we used Stata version 14.0 (StataCorp, Texas, USA). All other analyses were performed with IBM SPSS Statistics for Windows version 24 (IBM Corp., Armonk, USA) and Microsoft Office 365 Excel Version 1911.

## Results

### Comparison over two generations

**Fig. 1:**
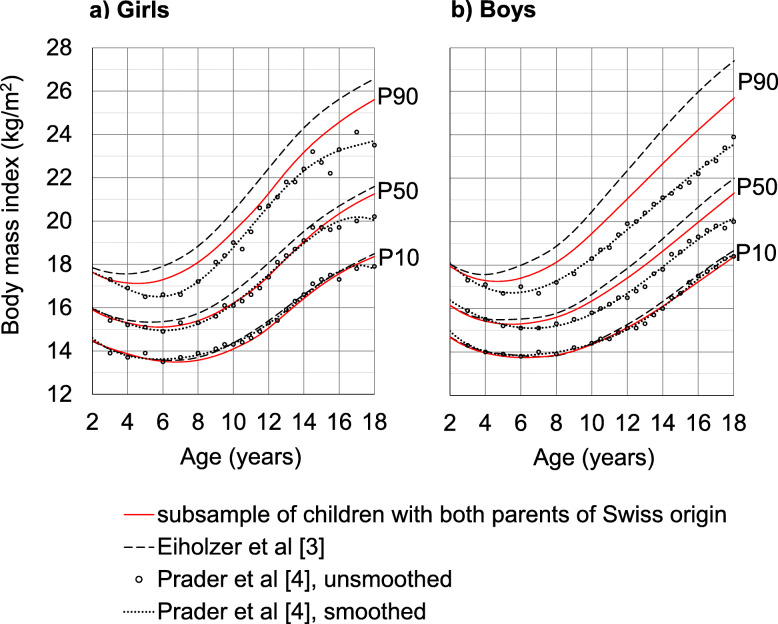
BMI-for-age percentiles of (**a**) girls and (**b**) boys with both parents of Swiss origin compared with the smoothed percentiles of Prader et al. [4]. For orientation, new BMI percentiles for Switzerland [3] were added

We specified the LMS models as 3/8/5 for girls and 3/6/4 for boys. Sex-specific values of P10, P50 and P90 are presented in Fig. 1.

On P10, girls today with Swiss parents still weigh the same as girls two generations ago. On P50, girls are not heavier until puberty, and then are about 1 kg/m^2^ heavier (equivalent to 2.8 kg at P50 height) by 17 years of age. On P90, girls above age 4 and onwards are now heavier than two generations ago with the biggest differences occurring at the age of 7 with 1 kg/m^2^ (1.5 kg) and from 15 years (2.7 kg) to 18 years (2 kg/m^2^, 5.5 kg). Fifteen and 6% of today’s proportion of girls lie above the Prader P90 and P97, respectively.

Similar trends on all percentiles were also observed for the boys with both parents of Swiss origin. On P50, boys are heavier by 0.7 kg/m^2^ from 11 years (1.5 kg) onwards to 17 years (2.2 kg). On P90, boys are significantly heavier and the difference increases with age from about 1 kg/m^2^ at 7 years to 2 kg/m^2^ at 18 years (6.4 kg at P50 height). Eighteen and 10% of today’s proportion of boys lie above the respective Prader P90 and P97.

### Differentiation by parent origin

Children of parents from Northern/Central Europe or Switzerland have lower rates of overweight and obesity than children whose parents originate from Southern Europe (Table 1).

**Table 1:** Rates of overweight and obesity (in percent) according to parent origin

Origin of both parents	Girls			Boys		
	n	Overweight^**a**^	Obese	n	Overweight^**a**^	Obese
**Switzerland**	2816	8.8	1.6	2867	9.6	1.2
**Northern/Central Europe**	383	7.0	0.8	387	5.4	1.0
**Italy, Spain, Portugal**	326	19.9	6.1	435	20.5	5.7
**Balkan region**	526	22.1	6.5	758	28.3	9.5
**Turkey**	135	23.0	5.2	144	25.0	6.3
**One parent from Switzerland**	1032	9.4	1.6	1084	9.0	1.6
other parent: Northern/Central Europe	312	8.3	0.6	348	7.5	1.7
other parent: Italy, Spain, Portugal	238	11.3	2.5	223	9.0	0.9
other parent: Balkan region	74	6.8	0	89	11.2	4.5
other parent: Turkey	22	13.6	0	22	18.2	4.5
other parent: from other regions	386	9.3	2.3	402	9.5	1.0
**Not from the same region/from other regions**	778	17.5	3.0	872	16.5	3.6
**Total**	5996	12.0	2.5	6547	13.4	2.9
**χ**^**2**^**-test**^**b**^		χ^2^(12) = 169.96; ***p*** < 0.001^d^			χ^2^(12) = 313.53; ***p*** < 0.001^d^	
**χ**^**2**^**-test**^**c**^		χ^2^(2) = 119.93; ***p*** < 0.001			χ^2^(2) = 217.51; ***p*** < 0.001	

^a^ Category including obese subjects defined by the International Obesity Task Force (IOTF) cut-offs [8]

^b^ χ^2^-test for association between IOTF category (non-overweight, overweight, obese) and origin of both parents (all categories)

^c^ χ^2^-test for association between IOTF category (non-overweight, overweight, obese) and origin of both parents (Switzerland versus Southern Europe)

^d^ One expected cell frequency was smaller than 5 (Northern/Central Europe)

**Fig. 2:**
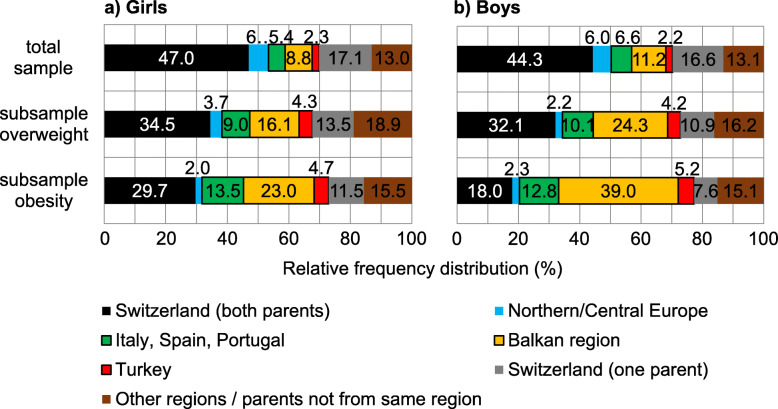
Distributions of origin in the categories of overweight (including obesity) (*n* = 1599) and obesity (*n* = 339) according to the International Obesity Task Force (IOTF) cut-off criteria [8] and in the contemporary core sample (*n* = 12,543, age: 2–19 years)

From our data set, 18% of children have parents from Southern Europe, but these children account for 29% (girls) and 39% (boys) of all children in Switzerland who are overweight and even make up 41% (girls) and 57% (boys) of all obese children (Fig. 2).

### Comparison of BMI percentile distributions by origin and between two generations

**Fig. 3:**
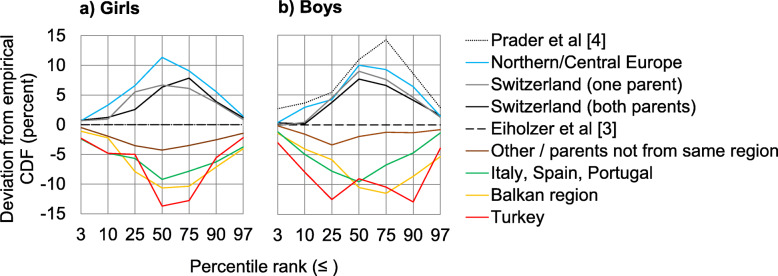
Deviations from the empirical cumulative distribution function (EDF) of percentile ranks compare the sex-specific subgroups and the Prader sample [4] to the sample of Eiholzer et al. [3], which is represented by the zero line. Lines above and below the zero line indicate a higher proportion of thinner and overweight children and adolescents, respectively

There was very little influence on the BMI percentile distribution when comparing children with both parents originating from Switzerland to children with only one parent originating from Switzerland (Fig. 3). Boys and girls with parents from Southern Europe are generally heavier based on the negative deviations over the whole range of percentile ranks. Compared to the entire sample, there are about 10% less children with Southern European origins below P50 and P75. Conversely, there are about 10% more children from Northern/Central Europe below P50.

Children and adolescents from the Prader sample with a BMI around P97 of our contemporary core sample were rare; BMI distribution deviations of the Prader sample show more children around P3 and none above P97. The proportion of girls with either one or two parents of Swiss origin at P50 is identical with the Prader sample (Fig. 3). Boys, irrespective of their parents’ origin, are heavier than two generations ago. At P50, the proportion of boys from the Prader sample is 10% higher than our contemporary core sample and 3% higher than boys with two parents of Swiss origin.

### Comparison of origin-specific P50 with national BMI percentiles

#### Italy, Spain, Portugal

**Fig. 4:**
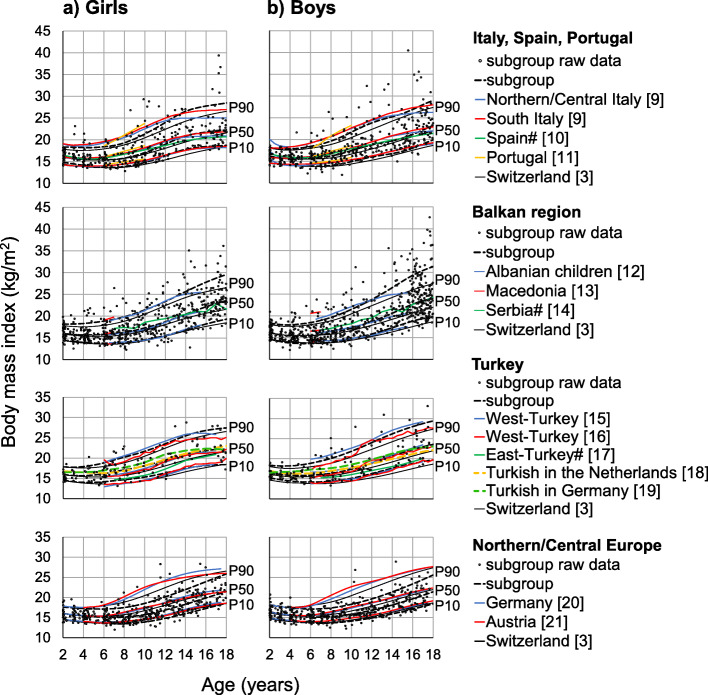
Raw subgroup data of children with parents from Italy/Spain/Portugal, Balkan region, Turkey, and Northern/Central Europe plotted against the new P10, P50 and P90 for Switzerland [3]. Group-specific P10, P50 and P90 are indicated as well as the established values for respective European countries/regions. Data are given as the arithmetic mean for references marked with #

We specified the LMS models as 3/7/3 for girls and 3/7/4 for boys. Up to 9 years, P50 of girls with both parents from Italy, Spain or Portugal resembles our contemporary P50 of Switzerland (Fig. 4). From age 10 onwards, P50 for this subgroup rises more steeply and becomes 1.5 kg/m^2^ higher at the age of 14 (3.9 kg at P50 height) and corresponds closely to P50 of girls from Southern Italy. P50 of boys with both parents from Italy, Spain or Portugal are similar to the contemporary P50 of Switzerland until 6 years only. Thereafter, P50 steeply rises and is about 1 kg/m^2^ higher from 10 years on, with a less emphasized difference at 15 years (2 kg at age 10 and 3 kg at age 18). P50 for this male subgroup is lower than P50 of Northern/Central and Southern Italy only until around the age of 17 years, where it then exceeds the published country-specific value.

#### Balkan region

We specified the LMS models as 3/5/3 for girls and 3/7/3 for boys. From the age of 4 years, P50 of girls with parents from the Balkan region is higher than the contemporary P50 of Switzerland (Fig. 4). Between 6 and 12 years, their P50 is about 0.5 kg/m^2^ higher (1 kg at P50 height) and at 18 years, this value is 2 kg/m^2^ higher (5.5 kg). P50 for this subgroup corresponds to P50 for both Albanian-born girls living in the Balkan region and Macedonian girls aged 6–7 years. For boys with parents from the Balkan region, their P50 corresponds up to 8 years with the contemporary P50 of Switzerland. At the age of 12 years, their P50 is 1 kg/m^2^ (2.3 kg) higher and at the age of 18 years, it is 2.2 kg/m^2^ (7 kg) higher. Their P50 is lower than P50 of Macedonian 6–7-year-old boys. From the age of 12, their P50 continually exceeds that of Albanian-born boys living in the Balkan region. For both girls and boys, there were more children above the country-specific P97 than the 3% expected.

#### Turkey

We specified the LMS models as 3/5/3 for both girls and boys. From our contemporary core sample, P50 of girls with parents from Turkey is higher than the contemporary P50 of Switzerland from 4 years of age; the maximum difference is 1.6 kg/m^2^ at 14 years (Fig. 4; 4.2 kg at P50 height). Their P50 also lies above P50 of Turkish girls. P50 of Turkish girls in the Netherlands is similar to children of Turkish origin in Switzerland, but P50 for girls with Turkish origin in Germany is higher. P50 of boys with Turkish parents is slightly higher than the contemporary P50 of Switzerland during childhood. From 11 years onwards, the difference exceeds 1 kg/m^2^ with a maximum of 1.8 kg/m^2^ at the age of 16 (5.5 kg at P50 height) and is similar to P50 of boys in Western Turkey. P50 of Turkish boys in Switzerland is similar to Turkish children in the Netherlands until the age of 12, and then Turkish boys in Switzerland are heavier. P50 of boys with Turkish origin in both Germany and Switzerland at the age of 16 is similar, but younger boys in Germany are heavier.

#### Northern/Central Europe

We specified the LMS models as 3/5/3 for both girls and boys. P50 of girls with parents from Northern/Central Europe corresponds up to the age of 5 with the contemporary P50 of Switzerland. Thereafter, their P50 is lower and the greatest difference of 1 kg/m^2^ (2.5 kg at P50 height) occurs at 10 to 15 years (Fig. 4). They are also less heavy compared to P50 of German girls with a maximum difference of 2 kg/m^2^ at the age of 13 years (5 kg) and to P50 of Austrian girls with a maximum difference of 1.8 kg/m^2^ at the age of 10 years (3.6 kg). The results for boys with both parents from Northern/Central Europe are similar. The greatest difference to the contemporary P50 of Switzerland is between 12 to 15 years with about 1 kg/m^2^ (2.3 to 3 kg). They are also less heavy compared to P50 of both German and Austrian boys, where the difference is approximately 1.8 kg/m^2^ at the age of 11 to 13 years (3.9 to 4.5 kg).

### Differentiation by socioeconomic position index

**Fig. 5:**
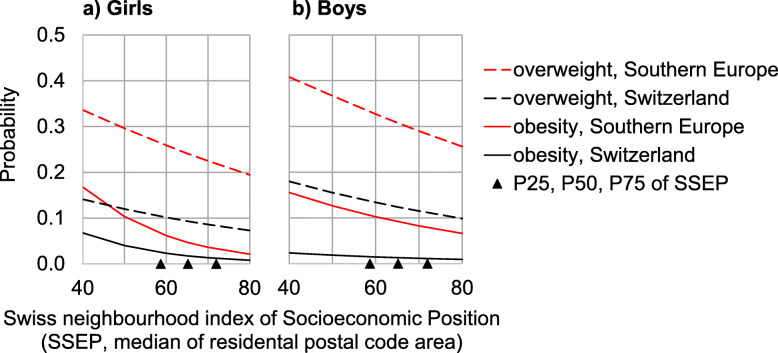
The relation between the Swiss neighbourhood index of Socioeconomic position (SSEP) and the chance of being overweight or obese for a. girls and b. boys with parental origin from Switzerland and from Southern Europe. The black triangles depict the 25th, 50th and 75th centiles of the observed SSEP

The SSEP index has little association with the probabilities of being overweight or obese (Table 2). The probability of being overweight is about 2.5 times higher for children whose parents both come from Southern Europe than for children with both parents coming from Switzerland (Fig. 5). The probability of being obese is 7-fold higher for boys and 2.7-fold higher for girls with parents from Southern Europe than for children with both parents coming from Switzerland.

**Table 2:** Logistic regression model coefficients for predicting overweight or obesity from origin^a^ and SSEP^b^

Model	Predictor	95% CI for regression coefficients β			95%CI for odds ratio			***p***-value	Model fit
		Lower	β	Upper	Lower	Odds e^**β**^	Upper		
**Overweight, girls**	origin	−1.38	− 1.13	−0.87	0.25	0.32	0.42	0.000	McFadden’s *R*^2^ = 0.05, LR X^2^(2) = 90.30, *p* = 0.000
	SSEP	−0.03	− 0.02	0.00	0.97	0.98	1.00	0.010	
	constant	−0.83	0.06	0.96	0.44	1.06	2.60	0.892	
**Overweight, boys**	origin	−1.35	−1.15	−0.94	0.26	0.32	0.39	0.000	McFadden’s *R*^2^ = 0.06, LR X^2^(2) = 144.04, *p* = 0.000
	SSEP	− 0.03	−0.02	− 0.01	0.97	0.98	0.99	0.003	
	constant	−0.41	0.32	1.06	0.66	1.38	2.88	0.387	
**Obesity, girls**	origin	−1.52	−1.02	−0.52	0.22	0.36	0.60	0.000	McFadden’s *R*^2^ = 0.06, LR X^2^(2) = 36.10, *p* = 0.000
	SSEP	−0.08	−0.06	− 0.03	0.92	0.95	0.97	0.000	
	constant	−1.12	0.63	2.38	0.33	1.88	10.81	0.479	
**Obesity, boys**	origin	−2.52	−2.04	−1.55	0.08	0.13	0.21	0.000	McFadden’s *R*^2^ = 0.12, LR X^2^(2) = 101.76, *p* = 0.000
	SSEP	−0.05	−0.02	0.00	0.95	0.98	1.00	0.043	
	constant	−2.18	−0.74	0.71	0.11	0.48	2.02	0.317	

^a^ Dichotomized into 1 = ‘both parents from Switzerland’ versus 0 = ‘both parents from Southern Europe’

^b^ Swiss socioeconomic position index, interval scale, median, 65.2 (interquartile range, 58.7–72.0)

*CI* confidence interval, *LR* X^2^ likelihood ratio chi-square

## Discussion

The BMI percentiles of Prader et al. [4] were recently compared with contemporary BMI references [3]. Two generations lie between these two data sets and the current BMI percentile curves, including all children of Switzerland, are higher, which signifies heavier children in present-day Switzerland. In Switzerland, we are in the privileged situation of having a compulsory population register, a statistical evaluation of immigrants and emigrants, and an area-based measurement of SSEP. This enables us to investigate the association of generational, migration-related and socioeconomic factors with the observed BMI.

To investigate differences in BMI between the two generations independent of migratory factors, we excluded children with parents of non-Swiss origin from the contemporary core sample and compared only the BMI percentiles of children with two parents of Swiss origin and the Prader BMI percentiles, which included only Swiss children. P10 and P50 for girls were practically the same between the two generations, while P90 increased. For boys, the whole distribution shifted in a skewed fashion. From 4 years of age, the heaviest 10% of children and adolescents are considerably heavier today compared to children two generations ago. This phenomenon of increasing skewness in the BMI distribution has already been described and is attributed to a distinct subgroup that is genetically susceptible to obesity [22]. The IOTF cut-off for obesity throughout childhood and adolescence lies close to P100 of the Prader sample. This implicates that there were very few obese children in the Swiss city of Zurich two generations ago, although one should keep in mind the uncertainty of P100 due to the small case numbers included in the Prader longitudinal study. Of today’s children with parents originating from Switzerland, 1.2 and 1.6% of girls and boys respectively, are obese according to the IOTF definition.

Boys and girls with parents originating from countries of Southern Europe have a 2.5-fold higher risk of being overweight. While we found the same ratio for obese girls, this risk was 7-fold elevated for boys with parents from Southern Europe. Of all obese children in Switzerland, 57% of boys and 41% of girls have parents from Southern Europe, although the part of this immigrant group in Switzerland is only 13%. This shows that the increase in BMI of children in Switzerland is mainly due to the immigration of persons from Southern Europe into Switzerland, and not from an increase in weight of the local population. If immigrants would not have been included in the recent calculations of BMI percentiles [3], the increase of P90 would be less than half compared to the Prader study.

Marked variations in overweight and obesity prevalence between children from Northern and Central Europe versus Southern European origins is in line with recent findings [1, 23, 24]. This is also consistent with findings in the adult population of Switzerland, where adults from Northern Europe had the lowest prevalence of overweight and obesity while people from Southern Europe showed the highest [25]. The origin of parents has been highlighted for its important role in the prevalence of overweight and obesity [2, 26], however these studies insufficiently classified their study populations into only Swiss and non-Swiss groups. Only one study, limited to school children living in the city of Geneva, examined the influence of individual national migrant groups and could highlight the strong influence of nationality on the prevalence of overweight and obesity [27]. Based on the fact that Europeans from both southern and northern regions are among the largest groups of Swiss immigrants, it is very important to acknowledge their proportions in study samples, especially when comparing the prevalence of overweight and obesity over a certain period of time. The Swiss BMI Monitoring working group, responsible for all epidemiological research on childhood BMI under governmental mandate, has yet to consider this fact. The group found a 4% decrease in the prevalence of overweight in children of non-Swiss origin (living in Zurich, Berne and Basel) between 2005 and 2017, and interpreted this trend as a positive development in prevention [26]. Nevertheless, it is highly probable that this change is due solely to changes in migration. For example, the percentages of children in the city of Zurich changed between 2005 and 2017 as follows: Switzerland + 13%, Northern/Central Europe + 4%, Balkan region − 11%, Italy/Spain/Portugal − 4%, and Turkey − 2% [28]. On the basis of the overweight rates per region of origin and the changing proportions of children per group of origin, we can calculate a decline in the overall overweight rate of 2.5% for all children or 3.5% for children of non-Swiss origin during this time period, which is exclusively due to the altered migration.

The exclusion of changes in national proportions of the different migrant groups leads to further incorrect weighting. Northern and Southern European immigrants belong to different social groups that have different functions within the Swiss economic system; the differences in levels of income and education between the two groups are substantial [29]. The immigrant population of Southern European people are mainly less qualified workers, while immigrants from northern countries are advantaged both in terms of an above-average proportion with a tertiary education and the countries’ socioeconomic situation. Due to this heterogeneity of the non-Swiss group, the simple differentiation between Swiss and non-Swiss children is inappropriate for further analysis of the impact of income and education on BMI.

From the logistic regression analysis in this study, socioeconomic differences practically vanish behind the region of parental origin. We think that people from the same region, who leave their country during a similar time period and probably for similar reasons, share many characteristics such as genetic traits, cultural habits, eating habits, level of education as well as values and attitudes. It is therefore plausible that the area of origin is more predictive of the risk of overweight or obesity than socioeconomic parameters alone.

How well do the new Swiss BMI curves fit children of immigrants from the various European regions? To help pediatricians interpreting the BMI of children with parents of non-Swiss origin, we provide graphs showing the BMI values of all respective children presented as data points in conjunction with the new Swiss percentiles [3] and those of the countries of parent origin. From the age of 6, the BMI P50 of children from Southern Europe lies above P50 of the new Swiss curves, yet largely corresponds to the P50 of their countries of origin, except for Turkey where large regional BMI differences exist. P50 of the Northern European countries lies below the P50 of the new Swiss curves as well as below the P50 of the countries of origin. This is probably due to the fact that this particular subgroup is advantaged both in terms of an above-average proportion with a tertiary education and the countries’ socioeconomic status.

The majority of our study participants live in the canton of Zurich. One could therefore assume that our BMI study is not representative of Switzerland as a whole. However, the main variable for BMI is the varying proportions of foreigner groups. In terms of the proportion of foreigners, the canton of Zurich, in fact, provides an optimal representation of the entire country because the proportions of multicultural diversity closely match the overall proportions distributed across all 26 cantons of Switzerland [30]. A number of cantons, particularly in the French speaking parts of Vaud and Fribourg, were difficult to study as they did not wish to participate in a national evaluation of anthropometric parameters in school children [2]. In the official Swiss statistics on population, 13% of children and adolescents aged 0 to 19 years come from Southern Europe [30]. The respective percentage from our contemporary core sample is 18%. The official Swiss statistic is based on nationality, whereas this study targeted the origin of the parents. The difference in percentages is based on the acquisition of Swiss citizenship. For example, in the canton of Zurich, 37% of Swiss citizenship was obtained by people originating from Serbia (including Montenegro and Kosovo), Macedonia, Croatia and Bosnia-Herzegovina between 1994 and 2009 [31].

### Limitations

Our study is restricted by the lack of information regarding the percentage of the Swiss group comprising second or higher generation immigrants. To date, there is an estimated 7.2% of second-generation immigrants and 0.3% of third-generation or higher immigrants from the Swiss population who are aged 15 years and older [32]. We assume that a proportion of our sample of children with both parents of Swiss origin are, in fact, children of second-generation immigrants who certainly have higher cultural and genetic diversity compared to the Prader sample. Furthermore, we do not have data on the length of residence of immigrant children and parents since arriving in Switzerland. Within the last 8 years, 70% of children of foreign nationalities living in Switzerland were born in Switzerland [33]. This suggests that the majority of the children in our sample have spent most of their childhood in Switzerland. This value is even higher (90 to 96%) for children with parents from the Balkan region (Kosovo, Serbia, Northern Macedonia) or Turkey.

## Conclusion

Immigration from South European countries accounts for the main part of the increase in BMI and the prevalence of overweight and obesity in Switzerland.

Many European studies only distinguish between natives and immigrants [2, 24, 26], but not all immigrants are alike. Immigrants from Southern Europe are mainly less qualified workers with a low educational status and there is already a higher BMI and an increased prevalence of overweight and obesity in their country of origin. Immigrants from Northern and Central Europe, on the other hand, are in higher economic positions and their BMI is comparable to Switzerland. Hence, a decreased percentage of the prevalence of obesity can also be caused by higher immigration from northern European countries.

Children with parents of Swiss origin serve as an adequate comparison group to the sample of Prader and colleagues. Among the Swiss, only the heaviest children (P90) have become heavier, otherwise the difference among the two generations is rather marginal. We could also show that socioeconomic differences in Switzerland are less important than national origin with regard to the risk of overweight and obesity. A differentiated view regarding origin is essential for countries with large migrant populations in order to better understand the mechanisms of the prevalence of overweight and obesity.

## Abbreviations

BMI: Body mass index
EDF: Empirical cumulative distibution function
IOTF: International Obesity Task Force
Pn: nth percentile (e.g. P50 = 50th percentile)
SSEP: Swiss neighborhood index of socioeconomic position
